# Deletion of Two Genes in *Burkholderia pseudomallei* MSHR668 That Target Essential Amino Acids Protect Acutely Infected BALB/c Mice and Promote Long Term Survival

**DOI:** 10.3390/vaccines7040196

**Published:** 2019-11-26

**Authors:** Kei Amemiya, Jennifer L. Dankmeyer, Sergei S. Biryukov, Sylvia R. Treviño, Christopher P. Klimko, Sherry M. Mou, David P. Fetterer, Preston G. Garnes, Christopher K. Cote, Patricia L. Worsham, David DeShazer

**Affiliations:** 1 Bacteriology Division, United States Army Medical Research Institute of Infectious Diseases, Frederick, MD 21702, USA; kei.amemiya.vol@mail.mil (K.A.); jennifer.l.dankmeyer.ctr@mail.mil (J.L.D.); sergei.s.biryukov.mil@mail.mil (S.S.B.); sylvia_trevino@comcast.net (S.R.T.); christopher.p.klimko2.ctr@mail.mil (C.P.K.); sherry.m.mou.civ@mail.mil (S.M.M.); christopher.k.cote.civ@mail.mil (C.K.C.); worsham@fred.net (P.L.W.); 2 Biostatistical Services, United States Army Medical Research Institute of Infectious Diseases, Frederick, MD 21702, USA; david.p.fetterer.ctr@mail.mil (D.P.F.); preston.g.garnes.ctr@mail.mil (P.G.G.)

**Keywords:** melioidosis, auxotroph, live attenuated vaccine

## Abstract

Melioidosis is an emerging disease that is caused by the facultative intracellular pathogen *Burkholderia pseudomallei*. It is intrinsically resistant to many antibiotics and host risk factors play a major role in susceptibility to infection. Currently, there is no human or animal vaccine against melioidosis. In this study, multiple *B. pseudomallei* MSHR668 deletion mutants were evaluated as live attenuated vaccines in the sensitive BALB/c mouse model of melioidosis. The most efficacious vaccines after an intraperitoneal challenge with 50-fold over the 50% median lethal dose (MLD_50_) with *B. pseudomallei* K96243 were 668 Δ*hisF* and 668 Δ*ilvI*. Both vaccines completely protected mice in the acute phase of infection and showed significant protection (50% survivors) during the chronic phase of infection. The spleens of the survivors that were examined were sterile. Splenocytes from mice vaccinated with 668 Δ*hisF* and 668 Δ*ilvI* expressed higher amounts of IFN-γ after stimulation with *B. pseudomallei* antigens than splenocytes from mice vaccinated with less protective candidates. Finally, we demonstrate that 668 Δ*hisF* is nonlethal in immunocompromised NOD/SCID mice. Our results show that 668 Δ*hisF* and 668 Δ*ilvI* provide protective cell-mediated immune responses in the acute phase of infection and promote long term survival in the sensitive BALB/c mouse model of melioidosis.

## 1. Introduction

Melioidosis is an emerging disease that is endemic in Southeast Asia and Northern Australia, and is caused by the Gram-negative, facultative intracellular pathogen *Burkholderia pseudomallei*. The organism can be found in soil and water and has been reported to be distributed in a much wider range of territory that includes areas of the Middle East, Africa, South America, and Central America [1]. Cases of melioidosis have occurred in these latter areas and it has been suggested that it has been under reported because of both lack of awareness of the presence of the pathogen and lack of clinical expectation of the disease.

In Northeast Thailand the mortality rate for melioidosis is greater than 40%, while in Northern Australia the mortality rate was reported to be 19% [2,3]. The initial introduction of *B. pseudomallei* may be through percutaneous inoculation of the skin or previous wound, ingestion of contaminated water or food, or inhalation of the organism. Pneumonia is the most common presentation of melioidosis, which suggest that inhalation may be a common route of infection with cases increasing during the rainy season. In addition, secondary pneumonia may occur from inoculation of the pathogen at a distal site. There are major risk factors in the host that influence the ability to develop melioidosis, with diabetes mellitus being the primary factor followed by excess alcohol consumption, renal disease, and lung disease [4,5]. In fact, it has been shown in a long-term study of melioidosis in Northern Australia that host risk factors correlate better with severity or outcome of melioidosis than the presence of specific pathogen virulence factors, such as the lipopolysaccharide O-antigen [6]. With the present increase of diabetes in the human population, this may portent the increase in cases of melioidosis in the near future. Due to the multiple routes of exposure, the potential involvement of *B. pseudomallei* strains with varying degrees of virulence, and different host susceptibility factors, melioidosis patients present with many different clinical manifestations that may cause diagnosis to be difficult or misleading [7,8]. Due to the potential use of *B. pseudomallei* as a biological agent, the Centers for Disease Control and Prevention in the United States considers *B. pseudomallei* (and *B. mallei*, the causative agent of glanders) as Tier 1 biological select agents.

Treatment of melioidosis with antibiotics may be difficult and require prolonged regimens because of the intrinsic resistance of *B. pseudomallei* to many antibiotics [9,10,11]. Currently, there is no human or animal vaccine against melioidosis; however, there are many preclinical vaccine platforms that show promise in mouse models of melioidosis. These include heat-inactivated whole cells of *B. pseudomallei*, *B. mallei*, or *B. thailandensis*, outer membrane vesicles, live attenuated strains, subunit vaccines (capsular polysaccharides, lipopolysaccharides, ATP binding cassette system protein, autotransporter protein, outer membrane protein, and type VI secretion system hemolysin co-regulated proteins), DNA vaccines, and glycoconjugate vaccines. A recent comprehensive review of these different vaccine platforms is discussed in Titball et al. 2017 [12]. Evaluation of preclinical melioidosis vaccines in small animal models has been conducted in BALB/c and C57BL/6 mice, with the latter model being the more resistant animal. Due to these differences, testing of melioidosis vaccines has been recommended by the Steering Group on Melioidosis Vaccine Development [13] in both mouse models. It was also suggested that vaccines against naturally acquired infection be evaluated by subcutaneous challenges compared to vaccines used for biowarfare purposes that would protect against infection through inhalation. Some of the important parameters of immune protection for both routes of infection would include protection against acute infection with some mediation against chronic infection, induction of sterile immunity after acute or chronic infection, an increase in both humoral (IgG, and possibly IgA) response, and cell-mediated (involvement of interferon (IFN)-γ producing T-cells) immune responses that might induce a longer lasting protective response [12].

With these vaccine parameters in mind, we created a group of live attenuated vaccines in *B. pseudomallei* MSHR668, a strain isolated from the blood of a melioidosis patient with encephalomyelitis in Australia, and evaluated them in the sensitive BALB/c mouse model of melioidosis. There were several reasons for choosing *B. pseudomallei* MSHR668 as the primary starting candidate. First, it is highly pathogenic in both BALB/c and C57BL/6 murine inhalation model of melioidosis [14]. Second, it has a type A lipopolysaccharide (LPS) O-antigen phenotype, which is the most common characteristic LPS in clinical melioidosis isolates [15,16,17]. Third, it is relatively easy to genetically manipulate as compared to other strains of *B. pseudomallei*. In this report as a proof of concept we focused on our studies on the creation and testing of MSHR668 auxotrophic mutants and characterized the protection provided by these strains in acute and chronic phases of infection in the sensitive BALB/c murine model of melioidosis. We also showed that the 668 Δ*hisF* histidine auxotroph was nonlethal in NOD/SCID mice, which are impaired in the development of T- and B-cells and have defective natural killer (NK) cells.

## 2. Materials and Methods

### 2.1. Bacterial Strains, Plasmids, Primers, and Growth Conditions

The bacterial strains, plasmids, and primers used in this study are described in Table 1. *Escherichia coli*, *B. thailandensis,* and *B. pseudomallei* were grown at 37 °C on LB agar (Lennox formulation) or in LB broth (Lennox formulation). When appropriate, antibiotics were added at the following concentrations: 100 μg of ampicillin (Ap), 100 μg of carbenicillin (Cb), 50 μg of trimethoprim (Tmp), 25 μg streptomycin (Sm), and 25 μg of kanamycin (Km) per mL for *E. coli* and 25 μg of polymyxin B (Pm) and 500 μg of Km per mL for *B. thailandensis* and *B. pseudomallei*. For induction studies, isopropyl-ß-D-1-thiogalactopyranoside (IPTG) was added to a final concentration of 0.5 mM. A 20 mg/mL stock solution of the chromogenic indicator 5-bromo-4-chloro-3-indolyl-ß-D-galactopyranoside (X-Gal) was prepared in *N*, *N* dimethylformamide and 40 μL was spread onto the surface of plate medium for blue/white screening in *E. coli* 10 G chemically competent cells. Amino acid auxotrophs were phenotypically assessed for growth on M9 Minimal Salts Medium containing 0.4% glucose with or without the relevant 40 μg/mL L-amino acid(s). All manipulations with *B. pseudomallei* were carried out in a class II microbiological safety cabinet located in a designated biosafety level 3 (BSL-3) laboratory.

### 2.2. DNA Manipulation

Restriction enzymes (Roche Molecular Biochemicals and New England BioLabs), Antarctic phosphatase (New England BioLabs, Ipswich, MA, USA), and T4 DNA ligase (Roche Molecular Biochemicals, St. Louis, MO, USA) were used according to the manufacturer’s instructions. When necessary, the End-It DNA end repair kit (Epicentre, Middletown, WI, USA) was used to convert 5′ or 3′ protruding ends to blunt-ended DNA. DNA fragments used in cloning procedures were excised from agarose gels and purified with a PureLink Quick Gel Extraction Kit (Invitrogen, Waltham, MA, USA). Bacterial genomic DNA was prepared from overnight LB broth cultures with the GenElute Bacterial Genomic DNA Kit (Sigma-Aldrich, St. Louis, MO, USA). Plasmids were purified from overnight LB broth cultures by using the Wizard Plus SV Miniprep DNA Purification System (Promega, Madison, WI, USA).

### 2.3. PCR Amplifications

PCR primers are shown in Table 1. PCR products were sized and isolated using agarose gel electrophoresis, cloned using the pCR2.1-TOPO TA Cloning Kit (Life Technologies, Waltham, MA, USA), and transformed into chemically competent *E. cloni* 10G. PCR amplifications were performed in a final reaction volume of 50 or 100 μL containing 1X FailSafe PCR PreMix D (Epicentre), 1.25 U FailSafe PCR enzyme mix (Epicentre), 1 μM PCR primers, and approximately 200 ng of genomic DNA. Colony PCR was utilized to screen for *B. pseudomallei* deletion mutants. Briefly, sucrose-resistant and Km-sensitive colonies were resuspended in 50 μL of water, and 5 μL was added to the PCR rather than purified genomic DNA. PCR cycling was performed using a Mastercycler pro S (Eppendorf) and heated to 97 °C for 5 min. This was followed by 30 cycles of a three-temperature cycling protocol (97 °C for 30 s, 55 °C for 30 s, and 72 °C for 1 min) and one cycle at 72 °C for 10 min. For PCR products larger than 1 kb, an additional 1 min per kb was added to the extension time.

### 2.4. Plasmid Conjugations

Mobilizable plasmids were conjugated to *B. thailandensis* and *B. pseudomallei* by using a membrane filter mating technique. Briefly, S17-1 harboring pMo130 and pBHR2 derivatives were inoculated into 3 mL of LB broth containing Km and Sm and grown at 37 °C for 18 to 20 h with shaking (250 rpm). *B. thailandensis* and *B. pseudomallei* were also grown under these conditions but without antibiotic selection. One hundred microliters of each saturated culture were added to 3 mL of sterile 10 mM MgSO_4_, mixed, and filtered through a 0.45 μm-pore-size nitrocellulose filter, using a 25 mm Swinnex filter apparatus (Millipore). Filters were placed on LB plates supplemented with 10 mM MgSO_4_ and incubated for 8 h in a 37 °C incubator. The filters were washed with 1–2 mL of sterile phosphate buffered saline (PBS) and 100–200 μL aliquots were spread onto LB agar plates containing Km and Pm and colonies were identified after 48 h incubation at 37 °C.

### 2.5. Construction of B. Pseudomallei Mutants

Gene replacement experiments with *B. pseudomallei* were performed using the *sacB*-based vector pMo130, as previously described [23]. Recombinant derivatives of pMo130 (Table 1) were electroporated into *E. coli* S17-1 (12.25 kV/cm) and conjugated with *B. pseudomallei* MSHR668 (or MSHR668 derivatives) as described above. Optimal conditions for resolution of the *sacB* constructs were found to be LB agar lacking NaCl and containing 10% (wt/vol) sucrose, with incubation at 25 °C for four days. *B. pseudomallei* deletion mutants were identified by colony PCR using the primers flanking the deleted regions of the targeted genes (Table 1). As expected, the PCR products generated from the mutant strains were smaller than those obtained from the wild-type strain.

### 2.6. Animals and Vaccinations

Female BALB/c mice 6–8 weeks old, which weighed between 18–22 g each, were obtained from the National Cancer Institute, Frederick, MD. For each study mice were each placed into groups of 14–15 mice and housed in separate pans of 10 mice and 4–5 mice unless otherwise stated. Female, 6–8 week old non-obese diabetic (NOD)/severe combined immunodeficiency (SCID) mice that are impaired in T-cell and B-cell development and deficient in natural killer cell function [31,32,33] were obtained from Charles River (Wilmington, MA) and placed into groups of 10 mice in separate containers. Mice were housed in clean, filtered top pans, with mouse litter, an enrichment item, and given water and food ad libitum, and mouse litter and pans were changed twice a week. Mice that needed to be vaccinated were vaccinated twice, three weeks apart, with 0.1 mL of the inoculum subcutaneously (s.c.) in the back of the mouse just off the central median line.

In the vaccine studies before challenge, a cohort of mice (3–5) from each study group was deeply anesthetized with anesthesia solution by intraperitoneal (i.p.) injection (0.1–0.2 mL) before exsanguination for sera and removal of spleens. All animal protocols and ethical evaluations had been considered and approved at USAMRIID before starting any proposed animal studies by the Institute IACUC committee, which issued the following statement: Research was conducted under an IACUC approved protocol in compliance with the Animal Welfare Act, PHS Policy, and other federal statutes and regulations relating to animals and experiments involving animals. The facility where this research was conducted is accredited by the Association for Assessment and Accreditation of Laboratory Animal Care, International and adheres to principles stated in the 8th Edition of the Guide for the Care and Use of Laboratory Animals, National Research Council, 2011.

### 2.7. Animal Challenges

For preparation of the inoculum for animal challenges, *B. pseudomallei* K96243 was grown for approximately 18 h in LB broth at 37 °C with shaking at 235 rpm. Dilutions of the culture were made in PBS, and the appropriate dilution was used for challenges. The final colony forming units (CFU)/mL was confirmed by plating aliquots of the diluted cultures. Mice were challenged four weeks after the second vaccination by i.p. injection of the inoculum in 0.1 mL. All animals after the challenge were observed at least twice daily for changes in their condition for up to 60 days unless stated. Animals that appeared to be moribund according to the intervention score developed by the Institute IACUC were humanly euthanized by CO_2_ inhalation or after anesthesia with a pentobarbital-based euthanasia solution given i.p. Animals left over from the studies were anesthetized and euthanized by CO_2_ inhalation. In some studies, mouse spleen and liver were removed for CFU quantitation. The limit of detection for residual organisms was 20 CFU/mL.

### 2.8. Antibody Determination

Antibody levels (class IgG, and subclasses IgG1 and IgG2a) were determined by ELISA in triplicate and performed at least once as previously described [34]. Briefly, irradiated-inactivated *B. pseudomallei* K96243 cells (10 μg/mL in 0.1 M carbonate buffer, pH 9.5) was used to coat a 96-well Immulon 2HB, round-bottom plate (Thermo-Fisher, Pittsburgh, PA) and incubated overnight at 4 °C. After washing and blocking (1 x PBS, 1% bovine serum albumin, 0.05% Tween 20) the antigen-coated plate, two-fold dilutions of sera was made in blocking solution in triplicate, and plates incubated for 1 h at 37 °C. After washing the plate 1/5000 diluted anti-IgG- (or –IgG1 or −IgG2a) horseradish peroxidase conjugate (Southern Biotechnology Associates, Inc., Birmingham, AL, USA) was added to the plate, and the plate was incubated for 1 h at 37 °C. The plate was washed and after color development, the plate was read at 450 nm with a reference filter of 570 nm. The results were reported as the reciprocal of the highest dilution giving a mean OD of at least 0.10, which was at least twice over the background signal ±1 SD. The results were combined with the results from the other mice in the same group, and the geometric mean with the geometric standard error of the mean of the group determined.

### 2.9. Spleen Cell Preparation

Splenocytes were prepared from vaccinated mice as previously described [34]. Briefly, spleens were removed from exsanguinated mice and disaggregated in wash medium (RPMI 1640 medium (Gibco, Thermo-Fisher, Grand Island, NY, USA) containing 25 mM HEPES and 2 mM glutamine) to make the extract. CFU were determined as needed on sheep blood or Trypticase Soy Agar plates (BD Diagnostics, Franklin Lake, NJ, USA) with undiluted extract or 10-fold dilutions in sterile water on triplicate SBA plates. The CFU were counted after 2–3 days of incubation at 37 °C. The limit of detection was 20 CFU/mL. The spleen homogenate was further treated with ACK Lysing Buffer (BioWhittaker, Walkersville, MD, USA) to lyse the red cells for 4–5 min, and then the homogenate was diluted with wash medium, and splenocytes pelleted by centrifugation at 1200 rpm for 10 min. The splenocytes were washed once and suspended in complete medium (wash medium containing 10% heat-inactivated fetal calf serum (Thermo-Fisher Life Technologies), 1 mM sodium pyruvate, 0.1 mM non-essential amino acids, 100 U/mL of penicillin, 100 μg/mL streptomycin, and 50 μM 2-mercaptoethanol), and the cells were counted with a TC20 automated cell counter (Bio-Rad, Hercules, CA, USA).

### 2.10. Stimulation of Splenocytes and interferon (IFN)-γ Expression

The induction of IFN-γ by antigen stimulated splenocytes was performed by a modification of a procedure previously described [35]. Briefly, 2 × 10^6^ splenocytes were incubated with either no antigen (media control), 2 × 10^7^ irradiation-inactivated *B*. *pseudomallei* K96243 cells, or PMA (100 ng/mL) with ionomycin (500 ng/mL) (Millipore-Sigma, St. Louis, MO, USA) in duplicate wells of a 48-well, flat-bottom, cell culture plate (Costar 3548, Corning, NY, USA) in a final volume of 0.5 mL of complete medium for 42–46 h at 37 °C with 5% CO_2_. After the incubation period, the cell-culture plate was centrifuged at 1200 rpm for 10 min, and the culture supernatant was collected and irradiated to sterilize the sample. IFN-γ was measured in the duplicate supernatant at least once using a Luminex MagPix (Themo-Fisher Life Technologies, Grand Island, NY, USA) as per the manufacturer’s directions using the Mouse Cytokine Magnetic 20-Plex Panel kit. The limit of detection for IFN-γ was approximately 5 g/mL.

### 2.11. Statistical Analyses

Continuous outcomes were log-transformed prior to analysis and summarized as geometric mean and geometric standard error, with comparisons made by a mixed model procedure applied to the log-transformed values. Numbers of animals surviving and times to death were compared by Fisher exact test or Log-Rank test, respectively. *p*-values of <0.05 were considered significant.

## 3. Results

### 3.1. Initial Vaccination Studies with Mutations Made in B. pseudomallei MSHR668

In preliminary studies, we examined a variety of attenuated mutants of *B. pseudomallei* MSHR668 (Table 1) to identify potential melioidosis vaccine candidates. We used this strain because it was easy to manipulate genetically [23] and was highly virulent by intraperitoneal or aerosol exposure [14,17]. At the same time we examined *B. thailandensis* E555, a strain that naturally acquired a capsular polysaccharide gene cluster that is similar to the cluster present in *B. pseudomallei* [24]. *B. thailandensis* E555 offered some protection when used as a whole-cell vaccine against a *B. pseudomallei* K96243 challenge under conditions similar to those used in this study to assess *B. pseudomalle*i MSHR668 live attenuated strains [36]. BALB/c mice were vaccinated twice s.c. with auxotrophic and/or attenuated mutants of *B. pseudomallei* MSHR668, *B. thailandensis* E555, and *B. thailandensis* E555 harboring the *B. pseudomallei* K96243 *fliC* gene (Table 1) [36,37]. This latter construct was included because it had been suggested that antisera against *B. pseudomallei* flagellin protein was partially protective against infection by *B. pseudomallei* in a diabetic rat model of melioidosis [37]. We hoped to see an enhancement of protection by wild-type *B. thailandensis* E555 or clearance of the challenge dose, but we saw neither with the *B. thailandesis* (pBHR2-Bp *fliC*) construct because the parent *B. thailandensis* E555 protected 100% of the challenged mice and appeared to have cleared the infection efficiently. Vaccinated mice were challenged four weeks later i.p. with 6.6 × 10^5^ CFU of *B. pseudomallei* K96243 (11 MLD_50_s) and observed for 30 days (Table 2). Two of the *B. pseudomallei* MSHR668 strains, 668 Δ*ppc (*aspartate auxotroph) and 668 Δ*gltB* (glutamate auxotroph) were not fully attenuated, and the mice did not survive after the first vaccination. Another group of mice vaccinated with the double mutant 668 ∆*gspD* ∆*syrF*, which harbored mutations in the genes encoding the type 2 secretion system (T2SS) outer membrane secretin protein (GspD), which was defective in the secretion of the innate immune response suppressor TssM [23], and a syrbactin-type proteasome inhibitor biosynthesis protein (SyrF), did not survive after the boost vaccination. *B. pseudomallei* 668 Δ*syrF* was only partially protective (2/9 survivors) and the spleens of the survivors were not sterile (Table 2). We do not know why the 668 Δ*gspD* Δ*syrF* double mutant was more pathogenic than the single 668 Δ*syrF* mutant, but it is possible that the Δ*gspD* mutation resulted in enhanced inflammation and death due to the absence of TssM secretion. Most of the mice that were vaccinated with 668 Δ*ilvI*, a branched chain amino acid auxotroph, and the double mutant 668 Δ*gspD* Δ*ilvI*, survived the challenge (10/10 and 9/10, respectively). Homogenized spleens from four survivors from each group were found to be sterile (Table 2). The two *B. thailandensis* strains, E555 and E555 (pBHR2-Bp*fliC*), were both protective with 9/9 survivors 30 days after challenge. However, the spleens of the survivors that we examined were not all sterile, with 3/4 and 1/4 containing viable bacteria, respectively. When the number of survivors in each vaccinated group was compared to the number of survivors in the PBS group, the differences were significant for all (*p* < 0.0010) except for the survivors vaccinated with 668 ∆*syrF* (*p* = 0.2580).

We next examined the murine antibody response to the live vaccines that we evaluated to determine if there was any correlation between the amount of protection offered by the vaccine and the antibody level. We used an ELISA assay with inactivated *B. pseudomallei* K96243 as the capture antigen to evaluate the class IgG and subclass IgG1 and IgG2a response to the vaccines. While most 668 ∆*ilvI*- and 668 ∆*gspD* ∆*ilvI*-vaccinated mice survived challenge, they exhibited the lowest IgG antibody titers (18,798 (2.82) and 39,102 (3.11), respectively) in the study except for the PBS control mice (Table 3). In contrast, the 668 ∆*syrF* vaccinated mice had the highest IgG titer of all the vaccine candidates, but they had the lowest number of survivors (2/10) (Table 2 and Table 3). Of the *B. thailandensis* strains examined, the E555 group had a much higher IgG (3-fold) titer than the E555 (pBHR2-Bp*fliC*) group, although both groups had a similar number of survivors (100%). In most cases, the IgG1 subclass titers were higher than the IgG2a subclass titers, except for the mice vaccinated with 668 ∆*syrF* (Table 3, IgG2a/IgG1 ratios). All antibody class and subclass titers were generally significantly greater (*p* < 0.05) than the PBS control mice, except for the mice vaccinated with 668 ∆*gspD* ∆*ilvI* mutant, which was due to the variability of antibody titers in this group of mice. In summary, the IgG class and subclass antibody responses to the live *B. pseudomallei* MSHR668 mutants and the *B. thailandensis* E555 derivatives did not correlate with the amount of protection provided (Table 2 and Table 3). 

### 3.2. Comparison of 668 ∆hisF, 668 ∆ilvI, and Other MSHR668 Deletion Derivatives as Live Attenuated Vaccines

**Figure 1:**
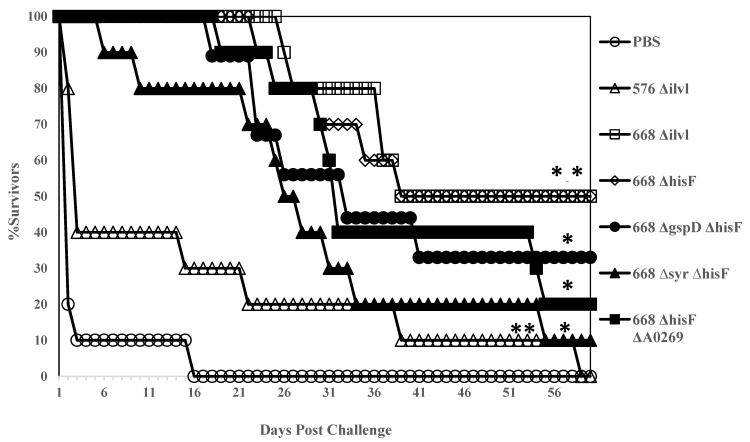
BALB/c mice vaccinated with 668 Δ*hisF* and 668 Δ*ilvI* are protected similarly during the acute phase of infection with *B. pseudomallei* K96243. Ten mice in each group were vaccinated twice (s.c.) with 1 × 10^6^ CFU in 0.1 mL of the respective mutant 21 days apart. Thirty days after the boost vaccination, the mice were challenged i.p. with 3.1 × 10^6^ CFU (50 MLD_50_s) of *B. pseudomallei* K96243, and challenged mice were followed for 60 days. Each group of 10 mice was tested twice in most cases, and the Figure is a composite of the survival of mice vaccinated twice with the respective whole cell vaccine before challenge. *p*-values comparing the mean survival time between the PBS control and other vaccines: * *p* = 0.0006; ** *p* = 0.0145 (576 Δ*ilvI*).

It was previously shown that a mutation in *hisF*, the gene encoding imidazole glycerol phosphate synthase in *B. pseudomallei* E8, caused histidine auxotrophy and virulence attenuation in BALB/c mice [38]. The *hisF* gene was mutated in *B. pseudomallei* MSHR668 by gene replacement (Material and Methods) and additional mutations (∆*gspD*, ∆*syrF*, and ∆*A0269*) were also incorporated into the 668 ∆*hisF* background (Table 1). *BURPS668_A0269* is the first gene in a cluster that encodes a terpherol-like compound that is a known inhibitor of phosphodiesterase 4 (PDE4) [39]. Since PDE4 is a key eukaryotic enzyme involved in the modulation of inflammatory mediators, we reasoned that elimination of the *B. pseudomallei* PDE4 inhibitor would increase the production of proinflammatory mediators such as IFN-γ. We also constructed an *ilvI* deletion mutation in *B. pseudomallei* 576, a strain that produces type B LPS, and compared it to the LPS type A strain 668 Δ*ilvI* (Table 1). In addition, we wanted to examine the protection afforded by Bp 576 with an *ilvI* mutation created by a deletion rather than a transposon insertion [19]. Interestingly, 576 Δ*ilvI* did not protect as well as 668 Δ*ilvI* against challenge with the type A LPS strain K96243 (Figure 1). Interestingly, 576 Δ*ilvI* did not protect as well as 668 Δ*ilvI* against challenge with the type A LPS strain K96243 (Figure 1). Mice vaccinated with these live attenuated strains (*n* = 10 for all groups) were followed for 60 days after challenge with 3.1 × 10^6^ CFU (50 MLD_50_s) of *B. pseudomallei* K96243 by the i.p. route of infection (Figure 1). All control mice (PBS) expired by day 16 post-challenge (MTD 3.5 days), and 7/10 mice vaccinated with strain 576 ∆*ilvI* were found dead or euthanized by day 15 post-challenge. No mice in the latter group were left after 60 days’ post-challenge (MTD 15.2 days). Both 668 ∆*ilvI* and 668 ∆*hisF* protected 100% of the mice during the acute phase of infection (21 and 25 days, respectively), and five mice from each group survived the challenge (MTD 46.6 and 45.2 days, respectively) through 60 days’ post-infection during the chronic phase of infection. At the same time, other 668 ∆*hisF* derivatives examined (668 ∆*gspD* ∆*hisF*, 668 ∆*syrF* ∆*hisF,* and 668 ∆*hisF* ∆*A0269*) did not protect mice during the acute phase of infection as well as the 668 ∆*hisF* or 668 ∆*ilvI* vaccinated mice (MTD 36.3 days, 33.5 days, and 34.0 days, respectively). After 60 days’ post-challenge, there were three, one, and two mice left in these groups, respectively. When spleens from all survivors were examined, they were all sterile except for the one survivor from the 688 ∆*syrF* ∆*hisF* vaccinated group. The results demonstrate that both 668 ∆*hisF* and 668 ∆*ilvI* provide highly sensitive BALB/c mice with long term sterilizing immunity against a significant i.p. challenge with virulent *B. pseudomallei*. Interestingly, 576 Δ*ilvI* 338 did not protect as well as 668 Δ*ilvI* against challenge with the type A LPS strain K96243 (Figure 1).

We next examined the antibody response against *B. pseudomallei* K96243 in sera from mice vaccinated with the various whole-cell vaccine candidates. ELISAs were performed on a cohort (*n* = 3 or 4) of each group that were vaccinated before the matching cohorts were challenged (Table 4). We found that the 576 ∆*ilvI* vaccinated mice, which were the least protected mice, generated the lowest IgG antibody response (2722 (3.91)). The next least protected mouse groups (668 ∆*gspD* ∆*hisF*, 668 ∆*syrF* ∆*hisF*, and 668 ∆*hisF* ∆*A0269*) had the highest IgG antibody titers (13,680 (2.47), 14,775 (2.55), and 23,926 (2.65), respectively). The two most protective candidate whole-cell vaccines, 668 ∆*ilvI* and 668 ∆*hisF*, had moderate levels of IgG (5350 (1.45) and 5668 (1.31), respectively) compared to the other vaccine groups in this study. The subclass levels of IgG1 and IgG2a varied widely between the vaccine groups (Table 4), and the ratio of IgG2a to IgG1 ranged from 0.17 (668 ∆*syrF* ∆*hisF*) to 2.51–2.52 (576 ∆*ilvI* and 668 ∆*gspD* ∆*hisF*, respectively). The IgG2a to IgG1 ratio of the most protective whole-cell vaccines were 1.19 (668 ∆*ilvI*) and 0.71 (668 ∆*hisF*). In summary, it appears that the most protective vaccine candidates induced a moderate IgG antibody response compared to the least protective vaccine candidates which generated either the lowest or highest antibody titers.

### 3.3. Inactivated Whole-Cell B. Pseudomallei Vaccine Candidates

**Figure 2:**
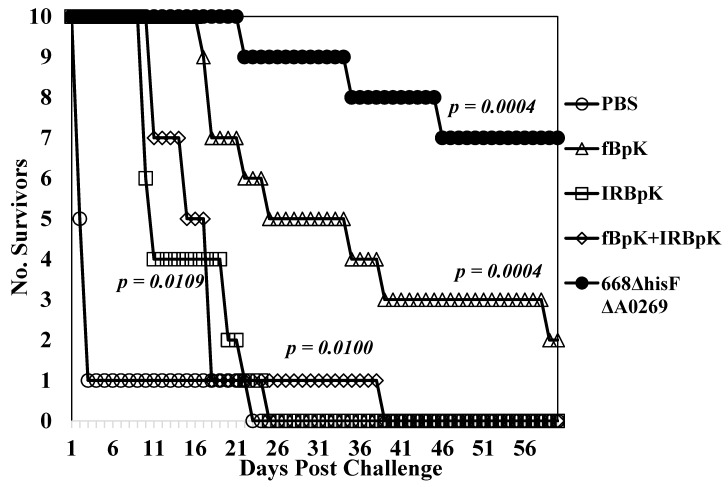
Inactivated *B. pseudomallei* K96243 (BpK) whole-cell vaccines do not protect mice as well as a MSHR668-derived auxotroph. Ten mice in each group were vaccinated with 50 µg of formalin-inactivated (fBpK), irradiated-inactivated (IRBpK), or a mixture of fBpK (50 µg) and IRBpK (50 µg) twice (s.c.) three weeks apart, and mice were challenged four weeks later (i.p.) with 2.5 × 10^6^ CFU (40 MLD_50_s) of K96243. At the same time, PBS and live attenuated 668 Δ*hisF* Δ*A0269* (1 × 10^6^ CFU) control groups were vaccinated twice (*n* = 10 in each group) and challenged at the same time. All mice were observed for 60 days after challenge. *p*-values comparing the mean survival time between the PBS control and other vaccines.

We next evaluated inactivated *B. pseudomallei* whole-cell vaccine candidates and compared them to one of the live attenuated vaccine candidates. We examined two types of inactivated *B. pseudomallei* K96243 preparations: Formalin-inactivated (fBpK) and irradiated-inactivated (IRBpK) preparations. BALB/c mice (*n* = 14) were vaccinated twice s.c. three weeks apart with 50 µg each of fBpK or IRBpK, and another group with 50 µg each of fBpK and IRBpK. For comparison, we vaccinated another group of mice with 1 × 10^6^ CFU of 668 ∆*hisF* ∆*A0269*. Four weeks after the boost vaccination mice (*n* = 10 for each group) were challenged i.p. with 2.5 × 10^6^ CFU (40 MLD_50_s) of *B. pseudomallei* K96243 and mice were observed for 60 days (Figure 2). The PBS control mice all expired by day 23 post-challenge (MTD 4.5 days) and the mice immunized with IRBpK expired by day 25 post-challenge, but there was a delay in the MTD (14.9 days) as compared to the control mice. In the group of mice vaccinated with fBpK, there was a further delay in MTD (29.1 days) with 20% of the mice surviving 60 days’ post-challenge (Figure 2). By comparison, 70% of the mice that received the live 668 ∆*hisF* ∆*A0269* vaccine survived the challenge for 60 days with a further delay in deaths (MTD 34.2 days). No CFU were detected in spleens from survivors in these two latter groups. Thus, there was a slight delay in deaths of mice that received the inactivated *B. pseudomallei* K96243 vaccine during the acute phase of infection as compared to the control PBS mice, with the fBpK vaccine being the best of those examined (100% survivors up to 16 days, post-infection). However, the live attenuated vaccine appeared to be much more protective through the acute (100% survival through 21 days’ post-infection) and chronic phase of infection (21–60 days’ post-challenge) than the inactivated *B. pseudomallei* vaccine candidates evaluated. The increased mouse survival afforded by 668 Δ*hisF ΔA0269* against K96243 challenge in this study (Figure 2) as compared to the initial study (Figure 1) could be due to the lower challenge dose used (40 MLD_50_s versus 50 MLD_50_s).

We measured the total IgG response and the IgG1 and IgG2a subclass responses generated by this group of vaccine candidates to IRBpK using ELISA (*n* = 4 for each group) (Table 5). Of the vaccine groups examined, we saw the lowest IgG response to the fBpK vaccine (1903 (1.19)), but the highest IgG murine response to the IRBpK vaccine candidate (75,361 (1.87)). The IgG titer to the mixture of the two inactivated vaccines was similar to the IRBpK vaccine alone (79,842 (1.38)). By comparison, the live 668 ∆*hisF* ∆*A0269* vaccine induced a moderate IgG response (8,016 (2.01)). All the IgG responses to the vaccines tested were significantly elevated compared to that of the PBS mice (*p* = 0.0053 - < 0.0001). The subclass IgG1 and IgG2a responses followed a similar pattern as the IgG titer, and of the vaccines examined in this study, with the live 668 ∆*hisF* ∆*A0269* which gave the closest IgG2a/IgG1 ratio to 1.0 of all the groups (0.38), whereas the other groups examined the IgGa/IgG1 ratio was less than the live vaccine (0.04–0.22).

We also used ELISA to compare the immune response to two immunogenic *Burkholderia* proteins that are exported by *B. pseudomallei* during infection. They were the hemolysin-co-regulated protein (Hcp1), which is associated with a functional type VI secretion system (T6SS-1) [27,28,40], and heat-shock protein 60 (GroEL), which is seen in melioidosis [41] and glanders [42] patients (see Table 5). We found a significant IgG response to Hcp1 in mice vaccinated with 668 ∆*hisF* ∆*A0269* vaccine strain (2834 (1.43), *p* = 0.0013), but not in mice vaccinated with inactivated strains of *B. pseudomallei* K96243 (50 (1.00) to 63 (1.18)). At the same time, we found a notable IgG response to GroEL in IRBpK (1008 (1.51)), fBpK + IRBpK (7551 (1.29)), and the 668 ∆*hisF* ∆*0269* (5053 (1.46)) vaccinated mice, but interestingly not in mice vaccinated with fBpK. Therefore, we saw an IgG antibody response to Hcp1 only in mice vaccinated with a live whole-cell vaccine, which suggests that T6SS-1 was active in the live attenuated mutant. However, unlike Hcp1, we saw an IgG immune response to GroEL in mice vaccinated with live and one type of inactivated (IRBpK) whole-cell vaccine. In this latter case, it is likely that GroEL is present in the IRBpK preparation that is recognized by the immune system of the vaccinated mouse.

### 3.4. Stimulation of Splenocytes from Whole-Cell Vaccinated Mice Shows the Development of a Cell-Mediated Acquired Immune Response to B. pseudomallei

**Figure 3:**
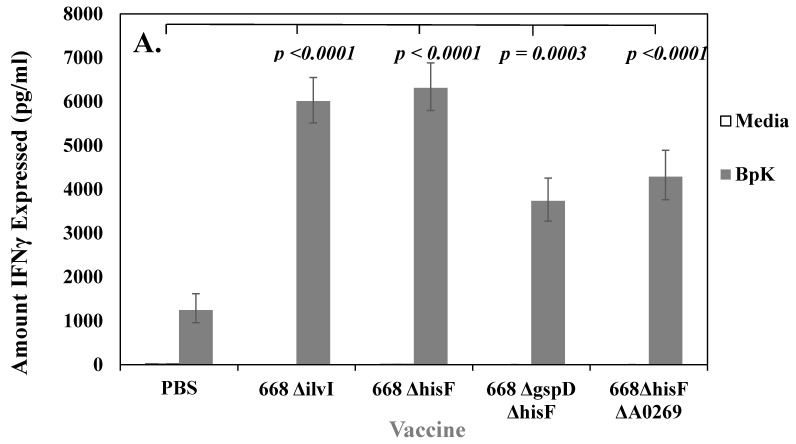

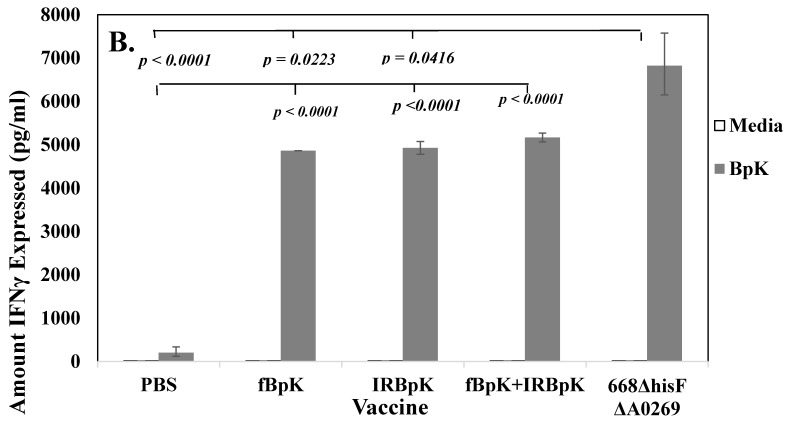
Cell-mediated immune responses were induced by both live and inactivated whole-cell vaccines, but live vaccines gave better protection in BALB/c mice. In most cases each vaccine was tested twice, and we show a representative of one study. N was 3–4 for each vaccine shown. (**A**) Splenocytes from mice vaccinated with live auxotrophs (1 × 10^6^ CFU) of 668 Δ*ilvI* and 668 Δ*hisF* and derivatives (as described in Figure 1) expressed interferon (IFN)-γ upon restimulation with IRBpK. (**B**) Splenocytes from mice vaccinated with 50 µg of formalin-inactivated BpK (fBpK), irradiation-inactivated (IRBpK, or a mixture of fBpK and IRBpK twice, 21 days apart (as described in Figure 2), and spleens were prepared from vaccinated mice four weeks after the second vaccination. Significant differences in the amount of IFN-γ expressed between the stimulated splenocytes from PBS vaccinated mice and stimulated splenocytes from test vaccines are shown in A and B. In B we also show a comparison between the stimulated 668 Δ*hisF* Δ*A0269* splenocytes verses other stimulated vaccines including the stimulated PBS control splenocytes.

We next wanted to determine if mice vaccinated with whole-cell vaccines developed acquired cellular immune responses that correlated with protection. We used ex vivo cultures of splenocytes isolated from vaccinated mice (*n* = 3 or 4, measured in duplicate) that were stimulated with IRBpK and measured the amount of IFN-γ produced in the culture supernatant after two days. IFN-γ has been shown to be required for survival against infection with *B. pseudomallei* in mice [43], and the production of IFN-γ by T cells was important in humans in addition to antibody responses for resistance to melioidosis [44]. In Figure 3, we show examples of the amount of IFN-γ produced after stimulation of splenocytes from BALB/c mice vaccinated with attenuated live (A) or inactivated (B) whole-cell vaccines. In both cases these vaccines were examined independently at least twice, and a representative example of the production of IFN-γ after restimulation of splenocytes with IRBpK is presented. In all cases, the live vaccines resulted in a significant difference (*p* < 0.0001) in the amount of IFN-γ expressed by splenocytes when they were stimulated with IRBpK over the same cells incubated with media only (Figure 3A). There was a similar amount of IFN-γ expressed by restimulated splenocytes from 668 Δ*ilvI* (6012 (1.09) pg/mL) and 668 Δ*hisF* (6316 (1.09) pg/mL) vaccinated mice that was significantly (*p* < 0.0001) higher than stimulated splenocytes from mice vaccinated with only PBS (1248 (1.30) pg/mL). In addition, we saw more IFN-γ expressed by restimulated splenocytes from 668 Δ*ilvI* and 668 Δ*hisF* vaccinated mice than restimulated splenocytes from 668 Δ*gspD* Δ*hisF* or 668 Δ*hisF* Δ*A0269* vaccinated mice although the differences in amounts were not significant (Figure 3A).

We also examined the amount of IFN-γ expressed by restimulated splenocytes from mice vaccinated with either inactivated fBpK, IRBpK, or both combined. The amount of IFN-γ expressed under these conditions were similar as shown in Figure 3B (4865 (1.00) pg/mL, 4928 (1.03) pg/mL, 5168 (1.02) pg/mL, respectively). Furthermore, the amount of IFN-γ expressed was higher than from stimulated splenocytes from mice vaccinated with only PBS, and in each case, the amount of IFN-γ produced was significantly higher (*p* < 0.0001) than when the same splenocytes were incubated in the presence of media only. Figure 3B also shows the comparison of IFN-γ expressed by restimulated splenocytes from mice vaccinated with a live vaccine as a control in the same study (668 Δ*hisF* Δ*A0269*), the amount was significantly higher (*p* < 0.0001) than that produced by splenocytes from mice vaccinated with the inactivated vaccine. Thus, we saw with both live and inactivated whole-cell vaccines that there was an acquired cellular immune response against *B. pseudomallei* K96243 in vaccinated BALB/c mice, however, only with the live attenuated vaccines did it appear that there was a correlation with the amount of protection when compared with control PBS mice (see Figure 1 and Figure 2).

### 3.5. B. pseudomallei 668 ∆hisF Is Highly Attenuated in NOD/SCID Mice

**Figure 4:**
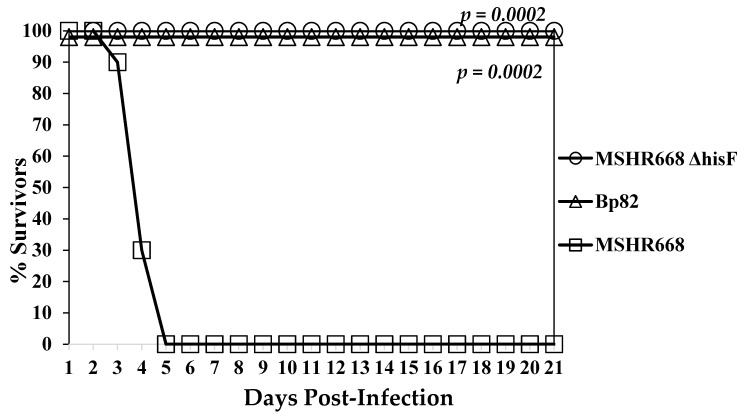
The 668 Δ*hisF* auxotroph was nonlethal in NOD/SCID mice. NOD/SCID mice (n = 10 for each group) that have impaired T- and B-cells and defective natural killer (NK) cells were inoculated (i.p.) with 668 Δ*his*F (10,270 CFU), Bp82 (Δ*pur*M) control group (11,330 CFU), and MSHR668 wild-type (12,870 CFU). After inoculation, mice were observed for 21 days. Comparison of mean survival time between MSHR668 and 668 Δ*hisF* or Bp82 was *p* = 0.0002.

We wanted to determine the degree of attenuation of the 668 ∆*hisF* strain, which led us to evaluate 668 ∆*hisF* in mice with a non-obese diabetic (NOD)/severe combined immunodeficiency (SCID) background. NOD/SCID mice are impaired in the development of T and B cells and have defective natural killer (NK) cells. We inoculated (i.p.) 10,270 CFU of 668 ∆*hisF* into NOD/SCID mice (*n* = 10), which was equivalent to 79 MLD_50_s for wild-type MSHR668 [17]. For comparison we inoculated 11,330 CFU of Bp82, which is a *B. pseudomallei* 1026b ∆*purM* adenine auxotroph that was shown to be severely attenuated [42], into another group of NOD/SCID mice (*n* = 9). As a control, we inoculated 12,870 CFU (equivalent to 99 MLD_50_s) of wild-type MSHR668 into another group of NOD/SCID (*n* = 10) mice and observed the mice for 21 days. As shown in Figure 4, mice inoculated with wild-type MSHR668 all expired by day 5 post-infection (MTD 4.2 days). On the other hand, all NOD/SCID mice inoculated with either 668 ∆*hisF* or Bp82 survived for 21 days post-challenge. When spleens and livers of the survivors were examined, 90% of the 668 ∆*hisF* inoculated mice were sterile (limit of detection 20 CFU/mL) and 100% of mice inoculated with Bp82 were found to be sterile. One of the mice infected with 668 Δ*hisF* harbored bacteria in the spleen and liver on day 21, but otherwise appeared healthy at the time of euthanasia. Thus, we demonstrated that 668 ∆*his*F is highly attenuated in immune defective mice.

## 4. Discussion

We have shown that two attenuated auxotrophs of MSHR668, 668 Δ*hisF* and 668 Δ*ilvI*, protected sensitive BALB/c mice during the acute phase of infection (up to 21 days post-challenge) in the murine model of melioidosis after a parenteral challenge of 50-fold over the MLD_50_ [17]. Interestingly, both amino acid auxotrophs protected the same number of mice in the acute phase and the chronic phase up to sixty days after the challenge, and survivors from both vaccinated groups cleared the challenge strain *B. pseudomallei* K96243. Furthermore, they induced a similar antibody IgG titer and cell-mediated immune response (IFN-γ expression) in BALB/c mice 30 days after the boost vaccination with the respective vaccines. The antibody titers induced by the auxotrophs did not correlate with protection, however, the cellular immune responses (IFN-γ expression) more closely correlated with protection during the acute phase of infection. Consequently, we examined the pathways for biosynthesis of L-histidine and the branched amino acids (BCAAs) L-isoleucine, L-leucine, and L-valine to see if there was a common factor between these two biosynthetic pathways that could be responsible for the similar host immune response to 668 Δ*hisF* and 668 Δ*ilvI* vaccines in the sensitive BALB/c melioidosis animal model.

There are 10 biochemical steps for biosynthesis of L-histidine [45,46]. The first step entails the condensation of phosphoribosyl pyrophosphate with ATP by ATP phosphoribosyltransferase (HisG/HisZ). L-histidine can feedback and inhibit this early step in the biosynthesis of L-histidine as well as can phosphoribosyl-ATP, AMP, or ADP [45,46]. The deletion in the *hisF* gene affects the synthesis of imidazole glycerol-phosphate synthase subunit, which is part of a heterodimer with the *hisH* product imidazole glycerol-phosphate synthase, glutamine amidotransferase subunit, in the fifth step of the pathway for the biosynthesis of L-histidine [45,46]. Importantly, a mutation in *hisF* can lead to at least two consequences for the bacteria: First is the lack of L-histidine for cellular protein synthesis and depression of the early pathway for L-histidine synthesis, and second is the lack of availability of 5-aminoimidazole-4-carboxamido-1-B-D-ribofuranosyl 5′monophosphate (AICAR) which is required for de novo purine biosynthesis [45,46,47,48]. It was found in *Salmonella typhimurium*, a Gram-negative, facultative intracellular pathogen, that a mutation in *hisF* affects the specific biosynthesis of ATP more than other nucleotides in the bacterium and can lead to purine auxotrophy [47]. 

The biosynthetic pathways for the BCAAs require only eight reactions for the synthesis of the three BCAAs [49]. The first common enzyme in the biosynthetic pathway of BCAA is acetolactate (or acetohydroxyacid) synthase (AHAS) of which there are three isozymes (AHAS I, II, and III) [49,50,51]. AHAS is involved in the decarboxylation of pyruvate and condenses the hydroxyethyl group with either another pyruvate molecule to give 2-acetolactate in the early pathway for valine or leucine biosynthesis or with 2-ketobutyrate to give 2-aceto-2-hydroxybutyrate in the early steps for isoleucine biosynthesis [50]. Each AHAS isozyme consists of a catalytic subunit and a regulatory subunit. Isozymes AHAS I and III are sensitive to feedback inhibition by valine. In most cases bacteria have a single AHAS isozyme which is frequently AHAS III [50]. In MSHR668, there appears to be only the isozyme AHAS III that consists of the gene product from *ilvI* (the catalytic subunit) and from *ilvH* (the regulatory subunit). In the AHAS III operon of both *B. pseudomallei* K96243 and MSHR668 there also appears to be a third gene (*ilvC*) that codes for a keto-acid reductoisomerase, which was pointed out previously [51]. The presence of AHAS III in *B. pseudomallei* K96243 was also reported by Kreisberg et al. [52] while looking for inhibitors for BCAA in pathogenic bacteria.

Hence, we found no apparent direct interaction of the L-histidine or BCAA biosynthetic pathways that could be responsible for the similar host immune response to the 668 Δ*hisF* or 668 Δ*ilvI* whole-cell vaccines, although we cannot discount secondary effects that might impact the expression of either biosynthetic pathway. However, the enzymes for the biosynthesis of these amino acids are found only in archaea, bacteria, fungi and plants, but not in mammals, which makes them potential targets for herbicides, fungicides and antimicrobial compounds [50,51,53]. This characteristic makes L-histidine and the BCAAs part of a group of nine amino acids (L-histidine, L-isoleucine, L-leucine, L-valine, L-lysine, L-methionine, L-phenylalanine, L-threonine, and L-tryptophan) that are unconditionally essential for mammalian cells for growth and multiplication, unlike nonessential amino acids that can be synthesized by mammalian cells [53,54,55]. Thus, we suggest that this common property of *hisF* and *ilvI* auxotrophs in the MSHR668 background may be responsible for the similar host immune response and makes them ideal whole-cell vaccines because they can mediate a protective humoral and cellular immune response in our mouse model of melioidosis. Mutations in *hisF* or *ilvI* may cause MSHR668 to be growth limited as well as replication deficient in the infected host which requires these essential amino acids to be supplied in their diet. Essential amino acids may not be at optimal levels within host cells, hence these conditions may augment auxotrophy of the vaccine strains [55]. Nevertheless, the auxotrophs can still maintain limited replication in the host, as we have shown for 668 Δ*hisF ΔA0269*, which exports Hcp1 by T6SS-1 in the vaccinated mouse (Table 5).

One factor that may have influenced the outcome of our murine studies was that we vaccinated mice s.c. with our live, whole-cells, and challenged the vaccinated mice by the i.p. route. We wanted to evaluate these candidates as potential vaccines similar to outer membrane vesicle (OMV) vaccines in which we could characterize protective host humoral and cell-mediated immune responses induced by our whole-cell candidate strains, in addition to still evaluate their attenuation [56,57,58]. In the OMV vaccine studies the particulate OMV were vaccinated s.c. into mice, and vaccinated mice were subsequently challenged by aerosol or intranasal (i.n.) exposure. It was not clear if we vaccinated mice by the i.p. route with our whole-cell vaccines rather than by i.p. that we would see a consistent protective immune response as we demonstrated with the 668 Δ*ilvI* and 668 Δ*hisF* auxotrophs. Furthermore, we could advance our best candidates to a higher animal model, such as a nonhuman primate, where the s.c. route of vaccination would most likely be used to initially evaluate the efficacy of the candidate vaccines as reported by Baker et al., (2017) [57] with their OMV vaccine. We choose to use i.p. challenges of vaccinated mice because we could better control the number of CFU delivered to each animal compared to aerosol exposure, and the method was much quicker and cost effective to perform when screening a large number of vaccinated animals under biosafety level three conditions. A comparison of protective immune responses to *B. pseudomallei* or *B. mallei* subunit candidate vaccines by i.p. or i.n. vaccination, and i.p. or i.n. challenges has been discussed previously [12].

Finally, we saw some variability in our antibody titers in groups of mice with our whole-cell vaccines. One factor that could influence the variability in the antibody titers in mice was the route of vaccination (s.c.). The variability in the titer could be because we did not use an adjuvant with our live, whole-cell vaccine to enhance the immune response to the vaccine similar to alum with subunit vaccines. However, with live, whole-cell bacterial vaccines we believe alum is not normally used. We have evaluated the effect of alhydrogel on the antibody and protective efficacy of the formalin inactivated whole-cell vaccine, but it did not enhance the antibody response nor the amount of protection over the vaccine without alhydrogel (Amemiya, unpublished). In fact, it decreased the antibody response to the vaccine and decreased the protective response to the vaccine. We do not know if we used i.p. vaccination that the antibody titers would be less variable with the whole-cell vaccines. Further studies (s.c. versus i.p.) would have to be carried out to rule out that the variable antibody response to the whole-cell vaccines was the result of the route of vaccination and not from differences in the live whole-cell vaccine strains.

## 5. Conclusions

In conclusion, our results with the Δ*hisF* and Δ*ilvI* mutants of MSHR668 show that some essential amino acid auxotrophs can mediate a protective immune response in the host during the acute phase of infection. This suggests that other essential amino acid auxotrophs of the host may also induce a protective humoral and cell-mediated immune response in animal models of melioidosis. Although it requires further studies, we believe that their effect would be in both the extracellular and intracellular stages of the pathogen in the host. For example, we postulate that they would be limited in their ability to replicate outside and within host cells, to mobilize host actin for motility, and to form host giant cells. Furthermore, because we have demonstrated this protection in the sensitive BALB/c mouse, we believe these types of essential amino acid auxotrophs could be even more protective in the acute/chronic phase of infection in the more resistant C57BL/6 murine model of melioidosis. We are pursuing further studies in the latter murine model of melioidosis with these specific auxotrophs.

## Figures and Tables

**Table 1:** Strains, plasmids, and primers used in this study.

Strain, Plasmid or Primer	Relevant Characteristics *	Source or Reference
*E. coli*		
*E. cloni*^®^10G	General cloning and blue/white screening	Lucigen
S17-1	*recA thi pro hsdR* RP42Tc::MuKm::Tn7 integrated into the chromosome; Sm ^r^, Tp ^r^, Pm ^s^	[18]
*B. pseudomallei*		
576 ∆*ilvI*	Original mutant was by transposon mutagenesis [19], for this study it was a derivative harboring a 1510-bp internal deletion of *ilvI* (∆*ilv*I)	[20]
K96243	Isolated in 1996 from a 34-year-old female diabetic patient in Khon Kaen, Thailand; Gm^r^, Km^r^, Sm^r^, Pm^r^	This study
MSHR668	Isolated in 1995 in Darwin, Australia from the blood of a 53-year-old male patient with severe melioidosis encephalomyelitis; Gm ^r^, Km ^r^, Sm ^r^, Pm ^r^	[21]
Bp82	1026b ∆*purM* derivative deficient in adenine	[22]
668 ∆*gspD*	MSHR668 derivative harboring a 1476-bp *gspD* in-frame deletion mutation (∆*gspD*)	[23]
668 Δ*ilvI*	MSHR668 derivative harboring a 1510-bp internal deletion of *ilvI* (∆*ilvI*)	This study
668 ∆*gspD* Δ*ilvI*	668 ∆*gspD* derivative harboring a 1510-bp internal deletion of *ilvI* (∆*ilvI*)	This study
668 Δ*syrF*	MSHR668 derivative harboring a 3463-bp deletion spanning *syrE* and 2608-bp at the 5′ end of *syrF* (Δ*syrF*)	This study
668 ∆*gspD* Δ*syrF*	668 ∆*gspD* derivative harboring a 3463-bp deletion spanning *syrE* and 2608-bp at the 5′ end of *syrF* (Δ*syrF*)	This study
668 Δ*gltB*	MSHR668 derivative harboring a 735-bp *gltB* in-frame deletion mutation (∆*gltB*)	This study
668 Δ*hisF*	MSHR668 derivative harboring a 65-bp *hisF* deletion mutation (∆*hisF*)	This study
668 ∆*syrF ΔhisF*	668 Δ*syrF* derivative harboring a 65-bp *hisF* deletion mutation (∆*hisF*)	This study
668 ∆*hisF ΔA0269*	668 Δ*hisF* derivative harboring a 1314-bp in-frame BURPS668_*A0269* deletion mutation (∆*A0269*)	This study
668 ∆*gspD ΔhisF*	668 ∆*gspD* derivative harboring a 65-bp *hisF* deletion mutation (∆*hisF*)	This study
668 Δ*ppc*	MSHR668 derivative harboring a 702-bp *ppc* in-frame deletion mutation (∆*ppc*)	This study
*B. thailandensis*		
E555	Isolated from soil in Cambodia in 2005; possesses a *B. pseudomallei*-like capsular gene cluster	[24]
Plasmid		
pCR2.1-TOPO	3931-bp TA vector; pMB1 *oriR*; Km ^r^, Ap^r^	Life Technologies
pCR2.1-Δ*ilvI*	pCR2.1-TOPO containing Δ*ilvI* PCR product generated with pDD120 [25] and primers ILV1-Nh and ILV4-Nh	This study
pCR2.1-*gltB*	pCR2.1-TOPO containing PCR product generated with MSHR668 gDNA and primers gltB-up and gltB-dn	This study
pCR2.1-Δ*gltB*	pCR2.1-*gltB* with a deletion of the 735-bp *Sal*I insert within *gltB*	This study
pCR2.1-*hisF*	pCR2.1-TOPO containing PCR product generated with MSHR668 gDNA and primers hisF-up and hisF-dn	This study
pCR2.1-*ppc*	pCR2.1-TOPO containing PCR product generated with MSHR668 gDNA and primers ppc-up and ppc-dn	This study
pCR2.1-*A0269*	pCR2.1-TOPO containing PCR product generated with MSHR668 gDNA and primers A0269-up and A0269-dn	This study
pCR2.1-Δ*A0269*	pCR2.1-*A0269* with a deletion of the 1314-bp *Nru*I fragment within *BURPS668_A0269*	This study
pCR2.1-Bp*fliC*	pCR2.1-TOPO containing PCR product generated with K96243 gDNA and primers BpfliC-up amd BpfliC-dn	This study
pMo130	Suicide vector for allelic exchange in *Burkholderia*; ColE1 *ori*, RK2 *oriT*, *xylE*, *sacB*, Km^r^	[26]
pMo130ΔNX	pMo130 digested with *Not*I and *Xba*I, blunt ended, and ligated to eliminate the *Not*I, *Pst*I, *Bam*HI and *Xba*I sites	[27]
pMo130-Δ*ilvI*	pMo130 containing *Nhe*I insert from pCR2.1-Δ*ilvI*	This study
pMo130-Δ*gltB*	pMo130 containing *Nhe*I insert from pCR2.1-Δ*gltB*	This study
pMo130-*hisF*	pMo130ΔNX containing *Nhe*I insert from pCR2.1-*hisF*	This study
pMo130-Δ*hisF*	pMo130-*hisF* digested with *Nru*I and *Not*I resulting in a 65-bp deletion within *hisF*	This study
pMo130-*ppc*	pMo130ΔNX containing *Nhe*I insert from pCR2.1-*ppc*	This study
pMo130-Δ*ppc*	pMo130-*ppc* derivative containing a deletion of the 702-bp *Pst*I insert within *ppc*	This study
pBHR2	Broad-host-range plasmid; Km^r^	[28]
pBHR2-Bp*fliC*	pBHR2 containing *Nhe*I insert from pCR2.1-Bp*fliC* in the *Xba*I site and oriented so that *fliC* is expressed from the constitutive Cm ^r^ promoter	This study
pEXKm5	Gene replacement vector for *B. pseudomallei*; Km^r^	[29]
pKOD	pEXKm5 harboring 1 kb of flanking DNA upstream and downstream of a 3463-bp deletion spanning *syrE* and 2608-bp at the 5′ end of *syrF*	[30]
Primers (5′−3′)		
ILV1-Nh	GCTAGCTTGAGCGCCAACGCCAATGC	eurofins
ILV4-Nh	GCTAGCAGACCGACCGTCACGTTCAC	eurofins
gltB-up	GCTAGCGCTCGATGGGCAACGATTCG	eurofins
gltB-dn	GCTAGCGCCATCTTGATCTGGATCTG	eurofins
hisF-up	GCTAGCTGGCTCTAGCTAAACGCATC	eurofins
hisF-dn	GCTAGCTCACAACCTCACTGCAATGC	eurofins
ppc-up	GCTAGCATCTCGCGAGCATCGATTTG	eurofins
ppc-dn	GCTAGCCAACCGCGAGATCGGTCTTC	eurofins
A0269-up	GCTAGCCAATTCGAAGGCGGTCGTG	eurofins
A0269-dn	GCTAGCGGCGCAGTTCCTTGTTCAGC	eurofins
BpfliC-up	GCTAGCGCTCACCGAACGATCGACAC	eurofins
BpfliC-dn	GCTAGCTTTGCTGCTGCGTCGTGCTG	eurofins

* bp: Base pairs; kb: Kilobases; r: Resistant; s: Susceptible; Sm: streptomycin; Gm: Gentamycin; Tp: Trimethoprim; Pm: Polymyxin B; Km: Kanamycin; Ap: Ampicillin.

**Table 2:** Evaluation of live attenuated *Burkholderia* strains as a potential melioidosis vaccines in a BALB/c murine model.

*Burkholderia* Strain	Amount	Vacination ^a^	Challenge Dose ^b^	No. Survivors	MTD	Sterility/Survivors
	Prime	Boost	(i.p.)	(After 30 Days)	(Days) ^c^	Examined ^f^
1. PBS control	na	na	6.6 × 10^5^	1/10	19.8	na
2. 668 Δ*ilvI*	4.7 × 10^5^	1.2 × 10^6^	6.6 × 10^5^	10/10	na	4/4
3. 668 Δ*gspD ΔilvI*	7.1 × 10^5^	4.2 × 10^5^	6.6 × 10^5^	9/10	29	4/4
4. 668 Δ*syrF*	2.3 × 10^5^	7.8 × 10^5^	6.6 × 10^5^	2/9^d^	22.3	0/2
5. 668 Δ*gspD ΔsyrF* ^e^	4.7 × 10^5^	9.9 × 10^5^	na	na	na	na
6. 668 Δ* ppc* ^e^	1.0 × 10^5^	na	na	na	na	na
7. 688 Δ* gltB* ^e^	1.0 × 10^5^	na	na	na	na	na
8. E555	3.7 × 10^6^	2.1 × 10^6^	6.6 × 10^5^	9/9 ^d^	na	3/4
9. E555 (pBHR2-BpfliC)	2.3 × 10^6^	2.2 × 10^6^	6.6 × 10^5^	9/9 ^d^	na	1/4

^a^ Mice were vaccinated s.c. twice for three weeks apart with the stated amount of whole cells in 0.10 mL; ^b^ Mice were challenged (i.p.) with 0.1 mL of *B. pseudomallei* K96243 four weeks after the boost vaccination; ^c^ Mean time to death (MTD) reported for animals that died; ^d^ One mouse from this group did not survive vaccination before the challenge; ^e^ Mice did not survive vaccination with these strains; ^f^ The limit of detection was 20 CFU/mL.

**Table 3:** Antibody response to potential live whole-cell melioidosis vaccines in BALB/c mice.

*Burkholderia* Strain ^a^	Antibody Titer ^b^			Ratio IgG2a/IgG1
	IgG	IgG1	IgG2a	
PBS control	50 (1.00) ^c^	50 (1.00) ^c^	450 (1.89)	na
668 Δ*ilvl*	18,798 (2.82) (*p* = 0.0288) ^d^	41,948 (3.22) (*p* = 0.0286)	17,523 (2.08) (*p* = 0.0202)	0.42
668 Δ*gspD Δilvl*	39,102 (3.11) (*p* = 0.0276)	37,144 (6.10)	8424 (2.64)	0.23
668 Δ*syrF*	2,528,449 (1.44) (*p* = 0.0009)	584,911 (1.44) (*p* = 0.0012)	1,756,334 (1.44) (*p* = 0.0011)	3
E555	194,704 (1.44) (*p* = 0.0016)	135,000 (1.89) (*p* = 0060)	125,843 (2.01) (*p* = 0.0040)	0.93
E555(pBHR2-Bp*fliC*)	64,901 (2.64) (*p* = 0.0175)	194,970 (2.64) (*p* = 0.0132)	39,102 (3.22) (*p* = 0.0421)	0.2

^a^*n* = 3 for each strain examined; ^b^ Antibody titers were reported as geometric mean with geometric standard error of the mean. Each titer was done in triplicate at least once; ^c^ The detection limit was 50, and we used 50 for statistical comparisons; ^d^ All *P* -values are relative to the phosphate buffered saline (PBS) control mice.

**Table 4:** Antibody response to attenuated *B. pseudomallei* MSHR668 Δ*hisF* and Δ*ilvI* strains and derivatives.

*B. pseudomallei* Vaccine Strain	Antibody Titer ^a^			Ratio IgG2a/IgG1
	IgG	IgG1	IgG2a	
PBS (4) ^b^	50 (1.00) ^c^	50 (1.00)	50 (1.00)	na
576 Δ*ilvI* (3)	2722 (3.91)	467 (1.93)	1170 (4.90)	2.51
668 Δ*ilvI* (4)	5350 (1.45) (*p* = 0.0009) ^d^	3377 (1.29) (*p* = 0.0003)	4016 (1.28) (*p* = 0.0003)	1.19
668 Δ*hisF* (4)	5668 (1.31) (*p* =0.0009)	6740 (1.59) (*p* = 0.0017)	4766 (1.65) (*p* = 0.0026)	0.71
668 Δ*gspD ΔhisF* (3)	13,680 (2.47) (*p* = 0.0247)	3175 (1.59) (*p* = 0.0112)	8000 (4.80)	2.52
668 Δ*syrF ΔhisF* (3)	14,775 (2.55) (*p* = 0.0256)	16,000 (2.25) (*p* = 0.0188)	2736 (2.57)	0.17
668 Δ*hisF ΔA0269* (4)	23,926 (2.65) (*p* = 0.0079)	5050 (*p* = 0.0015)	5350 (*p* =0.0320)	1.06

^a^ Antibody titers were determined in triplicate and done at least once and are reported as the geometric mean with the standard error of the mean shown in parentheses; ^b^ Number in parenthesis represents no. samples; ^c^ The detection limit was 50, and we used 50 for statistical purposes; ^d^ All *p*-values are relative to the PBS control mice.

**Table 5:** Comparison of the antibody response of inactivated and live *B. pseudomallei* vaccines in BALB/c mice.

Vaccine ^b,c^	Antibody Titer ^a^ (IRBpK)			Ratio IgG2a/IgG1	Antibody Titer ^a^	
					(Hcp1)	(Hsp60)
	IgG	IgG1	IgG2a		IgG	IgG
PBS	53.0 (1.06)	50.0 (1.00) ^d^	59.0 (1.12)	na	50.0 (1.00)	50.0 (1.00)
fBpK (50 μg)	1903 (1.19) (*p* <0.0001) ^e^	1008 (1.18) (*p* =0.0001)	224 (1.43) (*p* = 0.0292)	0.22	50.0 (1.00)	50.0 (1.00)
IRBpK (50 μg)	75,361 (1.87) (*p* = 0.0013)	142,544 (3.41) (*p* = 0.0074)	6362 (1.63) (*p* = 0.0017)	0.04	59.5 (1.19)	1008 (1.51) (*p* = 0.0049)
fBpK (50 μg) + IRBpK (50μg)	79,842 (1.38) (*p* = 0.0001)	63,496 (1.73) (*p* = 0.0029)	10,679 (1.65) (*p* = 0.0014)	0.17	63.0 (1.18)	7551 (1.29) (*p* = 0.0002)
668 Δ*hisF ΔA0269* (1 × 10^6^ CFU)	8016 (2.01) (*p* = 0.0053)	4490 (1.27) (*p* = 0.0002)	1692 (1.84) (*p* = 0.0107)	0.38	2834 (1.43) (*p* = 0.0013)	5053 (1.46) (*p* = 0.0149)

^a^ Antibody titers (class and subclass) are reported as the geometric mean with the geometric standard error of the mean against the corresponding antigen listed below. Significant differences (*p*-values) between the PBS titers and vaccine titers are listed below the antibody titers; ^b^ The following inactivated whole-cell vaccines were used: fBpK: Formalin-inactivated *B. pseudomallei* K96243; IR-BpK: Irradiation-inactivated *B. pseudomallei* K96243. All vaccines were given twice (s.c.), three weeks apart, and antibody titers were determined 28 days after the boost; ^c^ The numbers in parenthesis are the amounts of vaccine used or CFU used. N was equal to 4 for all vaccines evaluated, and antibody titers were done in triplicate at least once; ^d^ The detection limit was 50, and we used 50 for statistical purposes; ^e^ All *p*-values are relative to the PBS control mice.
